# Defining and Measuring Emergency Physician Productivity: Development of a Consensus‐Based Productivity Index

**DOI:** 10.1111/acem.70204

**Published:** 2025-12-08

**Authors:** Rohit Gandhi, Shawn Mondoux, Claudia Sauvé, John Boby Mesadieu, Jonathan Gravel

**Affiliations:** ^1^ Department of Family Medicine University of Ottawa Ottawa Ontario Canada; ^2^ Department of Emergency Medicine University of Ottawa Ottawa Ontario Canada; ^3^ Institut du Savoir Montfort Montfort Hospital Ottawa Ontario Canada; ^4^ Division of Emergency Medicine McMaster University Hamilton Ontario Canada

Emergency departments face growing pressure to improve efficiency amid rising patient volumes and wait times. Physician productivity has therefore become a key focus of quality improvement initiatives. Audit and feedback, which provide clinicians with performance data to guide practice change, are increasingly used. However, effective uptake requires measures that are clinically valid, actionable, and transparent [1]. A 2023 scoping review found wide variability in definitions and approaches, with no consensus on how to measure productivity [2]. Many departments still rely on crude metrics, such as patients seen per hour [3], which overlook patient complexity and operational constraints [4]. This limitation can obscure true performance and reduce the usefulness of feedback. More elaborate composite models exist, but few have been validated or widely adopted [5, 6].

This study aimed to develop a clinically valid, consensus‐based, multivariable productivity index reflecting the complexities of contemporary emergency medicine. Our approach combined physician‐prioritized metrics with system‐level modifiers, accounting for both patient complexity and operational constraints. We first conducted a scoping literature review to identify candidate measures. These measures were then refined using established consensus methods for performance indicator selection, including a modified Delphi process [7]. Finally, statistical modeling and collinearity testing were applied to select the most independent and meaningful predictors.

A total of 83 emergency physicians from 18 unique Canadian emergency departments participated in a modified Delphi process [7] to rate 34 candidate measures identified in the literature. Participants represented diverse practice settings including both academic and community hospitals, as well as urban and rural sites. Between November 2022 and August 2025, 18 virtual sessions were held. Each session included three to five participating physicians and consisted of two segments. In the first segment, participants rated each candidate measure on a Likert scale from 0 to 10, based on how likely the measure is to impact emergency physician productivity. In the second segment, participants were able to revise their ratings after discussion and group feedback to strengthen consensus. Nine additional measures were proposed by participants during the process, yielding a total of 43 measures (Table 1). All physicians present for each Delphi session completed both the initial and final rating rounds. Final ratings were calculated as mean scores, with variability assessed using the standard deviation.

**TABLE 1:** Final delphi ratings of candidate productivity measures and operational modifiers.

Metrics	Mean score ±Standard deviation	Modifiers	Mean score ±Standard deviation
Rate of patients seen per hour	8.51 ± 1.66	Rate of days with human resource shortages (nurses, technologists, support staff)	8.39 ± 1.48
Average length of stay (assessment → disposition)	7.55 ± 1.77	Average nurse order completion delay: time from physician order request to execution	8.38 ± 1.31
Average physician reassessment lag: time from nurse‐flagged reassessment to disposition decision	7.44 ± 1.69	Shift type: ambulatory, acute, or mixed	8.27 ± 1.53
Rate of patients seen per hour in second half of shift	7.18 ± 1.87	Proportion of CT/Ultrasound studies with order‐to‐report time greater than 3 h	8.09 ± 1.80
Average charting time per patient	6.95 ± 2.20	Average acuity score (marker of patient complexity)	7.79 ± 1.65
Consult‐to‐admission proportion: consultations resulting in hospital admission (> 24 h stay)	6.9 ± 2.02	Average admission‐to‐transfer time (marker of capacity/bed block)	7.83 ± 1.83
Imaging utilization rate (CT, Ultrasound, X‐ray per patient)	6.89 ± 1.90	Rate of patients requiring admission (marker of patient complexity)	7.76 ± 1.44
Rate of handovers given to colleagues per patient	6.56 ± 2.09	Patient availability/readiness: proportion of patients roomed and prepared for assessment	7.73 ± 1.54
Rate of initial assessments performed per hour	6.53 ± 2.48	Rate of handovers received per patient	7.51 ± 1.87
Rate of return visits within 72 h per patient	6.53 ± 2.10	Availability of essential equipment (e.g., otoscope)	7.5 ± 1.52
Consultation rate (requests per patient)	6.44 ± 2.15	Crowding: average number of patients awaiting assessment during physician shift	7.43 ± 1.74
Resource utilization rate: average number of orders per patient	6.38 ± 1.79	% Patients with disability or impairment (marker of patient complexity)	7.41 ± 1.89
Rate of patients discharged from waiting room during bed block	6.3 ± 2.00	Average consultant time delay: time from consultant request to consultant disposition	7.41 ± 1.93
Non‐basic lab utilization rate: proportion of patients receiving ESR, CRP, TSH, BNP, or Procalcitonin	5.24 ± 2.44	% Patients unilingual non‐English/French speaking (marker of patient complexity)	7.38 ± 2.06
		Average nursing staff experience/seniority	7.37 ± 1.34
		% Patients from nursing homes (marker of patient complexity)	7.35 ± 1.69
		Average patient age (marker of patient complexity)	7.18 ± 1.83
		Proportion of shifts with learners (students and residents)	6.81 ± 1.90

Based on Delphi session ratings, we standardized and consolidated time‐based metrics to enhance interpretability and model parsimony. Charting time, physician reassessment lag, and radiology delays were considered collectively as components of length of stay (LOS), informed by clinical relevance and collinearity testing as to avoid double‐counting overlapping constructs. LOS was defined as the interval from physician assumption of care to disposition decision. Measures that could not be reliably assessed, such as patient readiness in rooms or equipment availability, were excluded.

Next, we applied regression modeling to identify independent predictors of physician productivity, using 16 months of data (January 2022–April 2023) from 34 emergency physicians at a high‐volume urban community hospital. Correlation analysis was performed to detect interdependencies, and highly correlated measures were pruned in favor of clinical relevance and operational feasibility. For example, the Canadian Triage and Acuity Scale (CTAS), a five‐level scale used at triage in Canadian emergency departments, correlated strongly with patient age (r = −0.78), so only CTAS was retained. The final model explained 58% of the variance in physician productivity, supporting the selection of independent, meaningful predictors. The regression model formed the basis of our productivity formula, detailed below.

Productivity=Patients Seen+Physician Initial Assesments7÷Adjusted Work HoursLOS−Nursing Delay×CTAS Score

The numerator combines physician‐controlled metrics. Total patients seen is captured by adjusted patients per hour, which includes weighted physician initial assessments. Physician initial assessments are brief evaluations performed by physicians to initiate treatment and/or diagnostic testing before a full assessment. They have become increasingly common in Canada as a stopgap measure for long wait times. The divisor of seven reflects the empirical observation that seven physician initial assessments take approximately the same time as a complete patient encounter. Within adjusted work hours, ambulatory and walk‐in zone hours were weighted double to reflect the empirically observed increase in throughput compared with acute and resuscitation zone hours.

The denominator represents physician‐attributable encounter time measured as median LOS minus nursing delay to account for factors beyond physician control. Nursing order completion delays were adjusted using stepwise percentage reductions: no adjustment for delays ≤ 5.5 min (20th percentile), 12% reduction for 5.5–19.0 min (20th–80th percentile), and 20% reduction for > 19.0 min (80th percentile). These thresholds were empirically derived, and sensitivity analyses using alternative reductions showed minimal impact on physicians' relative productivity rankings, indicating stable results. LOS minus nursing delay is placed in the denominator to normalize productivity per unit time, reflecting the fact that longer LOS reduces throughput. Finally, adjusting for average CTAS allows fair comparisons across physicians by reflecting patient complexity. Using median LOS minimizes the influence of extreme values, while using the average CTAS provides a stable measure of patient acuity.

To validate our productivity index, we assessed reliability, robustness, and temporal stability. Test–retest reliability demonstrated week‐to‐week consistency in individual physician scores, indicating stable results over repeated measurements (*r* = 0.647, *p* < 0.001). Discriminant validity was supported by weak correlations with unrelated measures not included in the index, such as imaging ordering rates. Factor analysis confirmed coherent grouping of components, indicating that the selected measures captured most of the meaningful variation in productivity (88.6% of total variance). Known group testing showed that the index meaningfully differentiated physicians by patient volume (F = 42.4, *p* < 0.001, η^2^ = 0.98) and acuity (F = 8.8, *p* < 0.001, η^2^ = 0.90). Sensitivity analyses with alternative nursing delay penalty scenarios produced stable results (*r* = 0.968–0.971). Temporal validation showed consistent rankings across 2.5 years of empiric data (*r* = 0.936). These results indicate that the index is statistically robust, clinically interpretable, and stable across varying time and practice settings. When applied to our 16‐month, single‐site dataset, the index demonstrated substantial variability across physicians. Across the 35 physicians, mean productivity was 0.73 (SD 0.53), with individual averages ranging from 0.18 to 3.33. The distribution was right‐skewed (median 0.64; IQR 0.51–0.76), reflecting a central cluster of scores alongside distinct lower‐ and higher end values. These results illustrate that the index produces a clinically plausible spread of performance values that can support meaningful differentiation in audit and feedback.

When compared with other published productivity indices [5, 8], we believe our formula is more easily interpretable due to its transparent structure and use of clinically intuitive and directly measurable components. Robinson and colleagues used a large, real‐world EMR dataset and robust propensity‐score methods to compare attending physician productivity when working solo versus with residents [5]. This work is valuable, particularly for evaluating different staffing models and the impact of learners on flow. Our index advances this work by incorporating clinician‐prioritized metrics and system‐level modifiers, with the added ability to capture operational complexities. We believe this approach enhances both practical utility and fairness of performance assessment.

In 2025, Diercks and colleagues proposed a data envelopment analysis (DEA) framework to benchmark relative efficiency among emergency physicians, incorporating case‐mix adjustments and workload differences [8]. This approach more effectively accounts for patient complexity and provides a useful method for comparing physicians within a given practice setting. Our index builds on this work by including operational modifiers, such as nursing delays and shift type. Unlike DEA frameworks, it produces an absolute, directly interpretable score, which could better support operational planning, workload distribution, and cross‐site benchmarking once externally validated in other settings. By incorporating clinician‐prioritized metrics and distinguishing physician output from system‐level modifiers [9], our index allows fairer comparisons across diverse clinical contexts, including short‐staffed environments with varying patient acuity. In addition, involving physicians throughout development helps foster greater engagement and adoption [10]. These features strengthen both face validity and potential practical utility. The index can identify high performers and those needing targeted support or coaching, supporting integration into broader audit and feedback systems.

This study has several limitations. First, our study was conducted across three provinces and 18 Canadian emergency departments, which may limit international applicability. Nevertheless, the core measures of the index itself—including patients seen, shift hours, LOS, nursing delays and acuity scores—are common across most emergency care settings, supporting potential applicability elsewhere. Further validation in diverse healthcare systems is needed to confirm external validity. Second, although all measures could be differentially weighted, most index components were intentionally left unweighted to preserve interpretability and transparency. Third, this study focused solely on productivity. Other important performance aspects, such as quality of care and patient safety, are beyond its scope. The index is intended to serve as a complementary tool within broader audit‐and‐feedback frameworks. Finally, physician initial assessments, part of the physician‐controlled metrics in the numerator, are not routinely performed at all sites. In such cases, the index remains calculable, as these assessments primarily capture time spent on brief evaluations and help to avoid penalizing physicians in settings that do not rely solely on traditional patient encounters.

In conclusion, our consensus‐based productivity index provides a transparent, practical, and clinically grounded tool for assessing emergency physician performance. It accounts for physician‐controlled metrics while adjusting for system‐level and patient‐level modifiers beyond physician control. Future work should focus on external validation, routine implementation, and evaluation of its impact on physician performance and operational outcomes.

## Author Contributions

Study Concept and Design: R.G., S.M., C.S. Acquisition of Data: R.G., C.S. Analysis and Interpretation of Data: R.G., J.G. Drafting of the Manuscript: R.G., J.G. Critical Revision of the Manuscript: R.G., J.G., S.M. Statistical Expertise: J.B.M. Acquisition of Funding: R.G., C.S.

## Funding

This research has been funded by the Innovation Fund of the Alternative Funding Plan for the Academic Health Sciences Centers of Ontario. AHM‐21‐005.

## Conflicts of Interest

The authors declare no conflicts of interest.

## Data Availability

The data that support the findings of this study are available from the corresponding author upon reasonable request.
